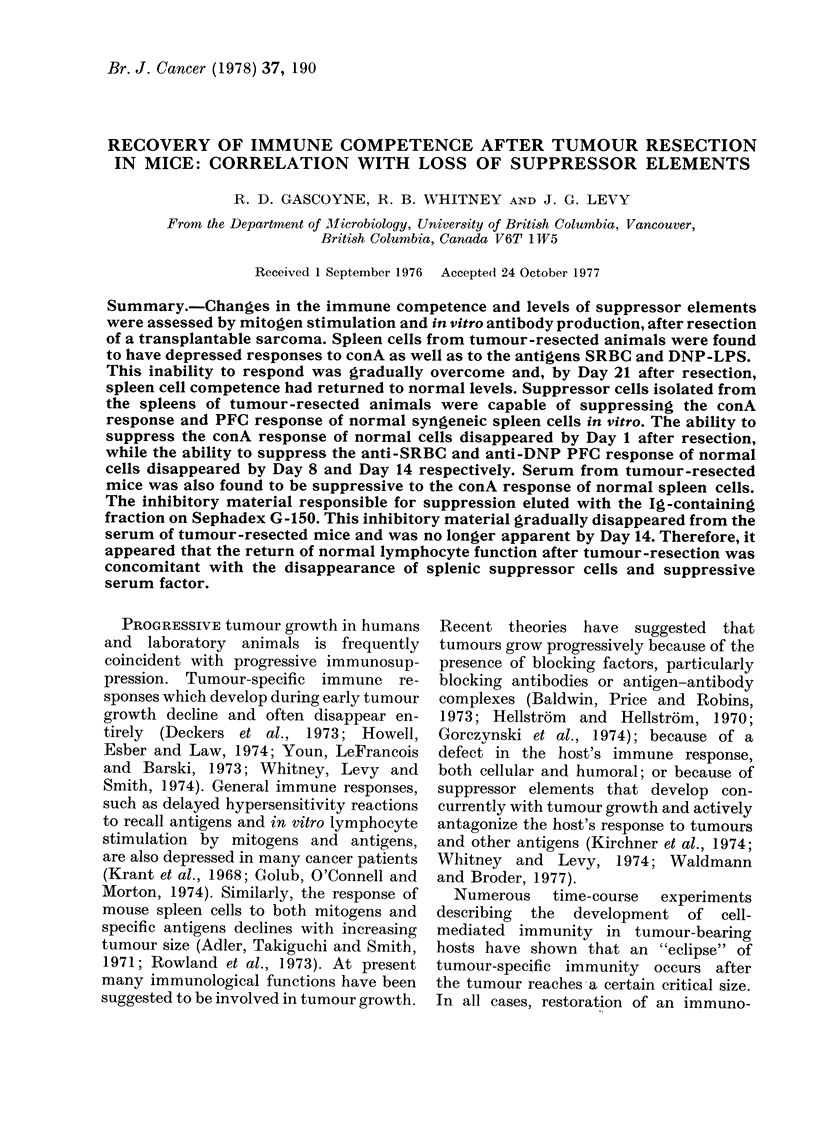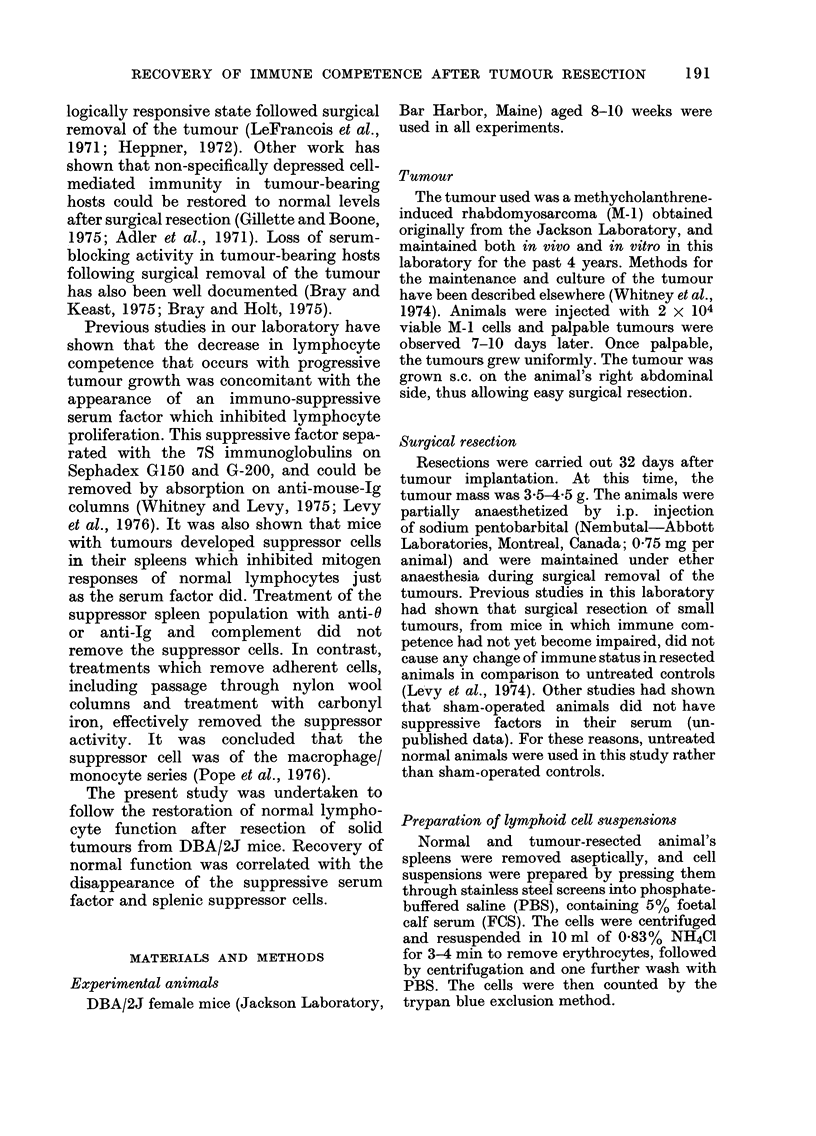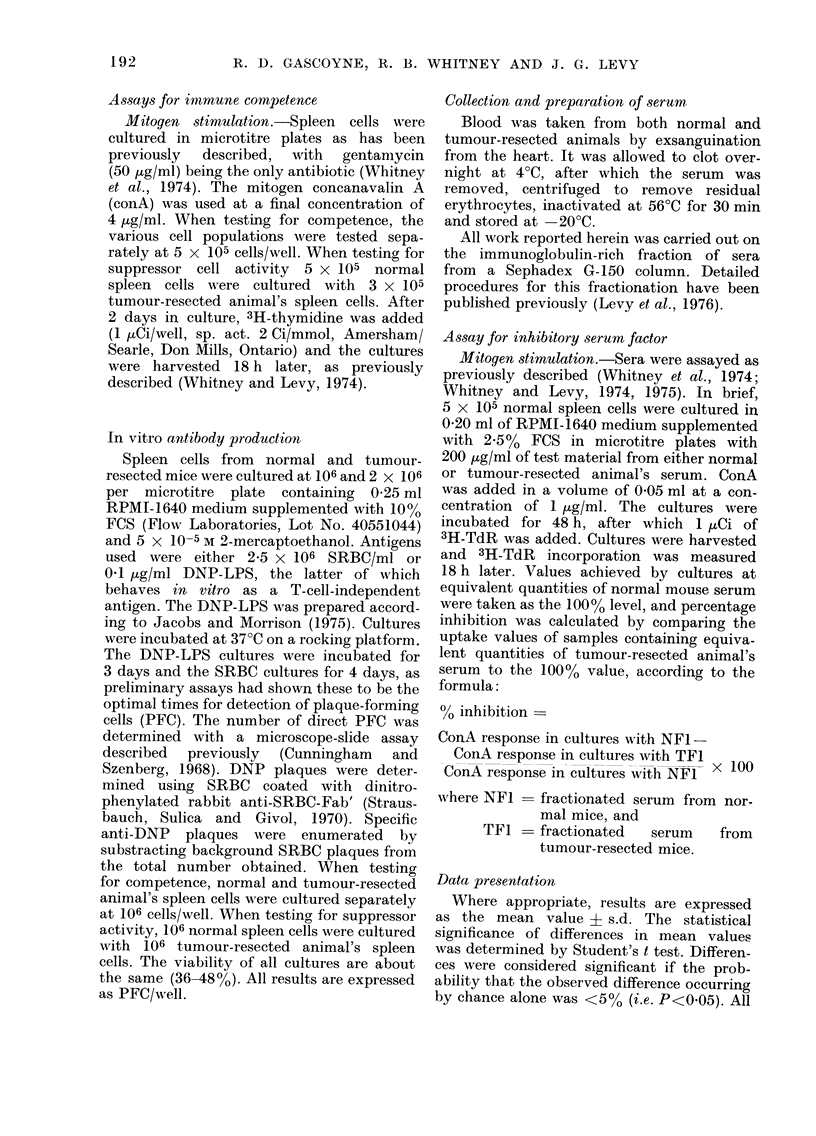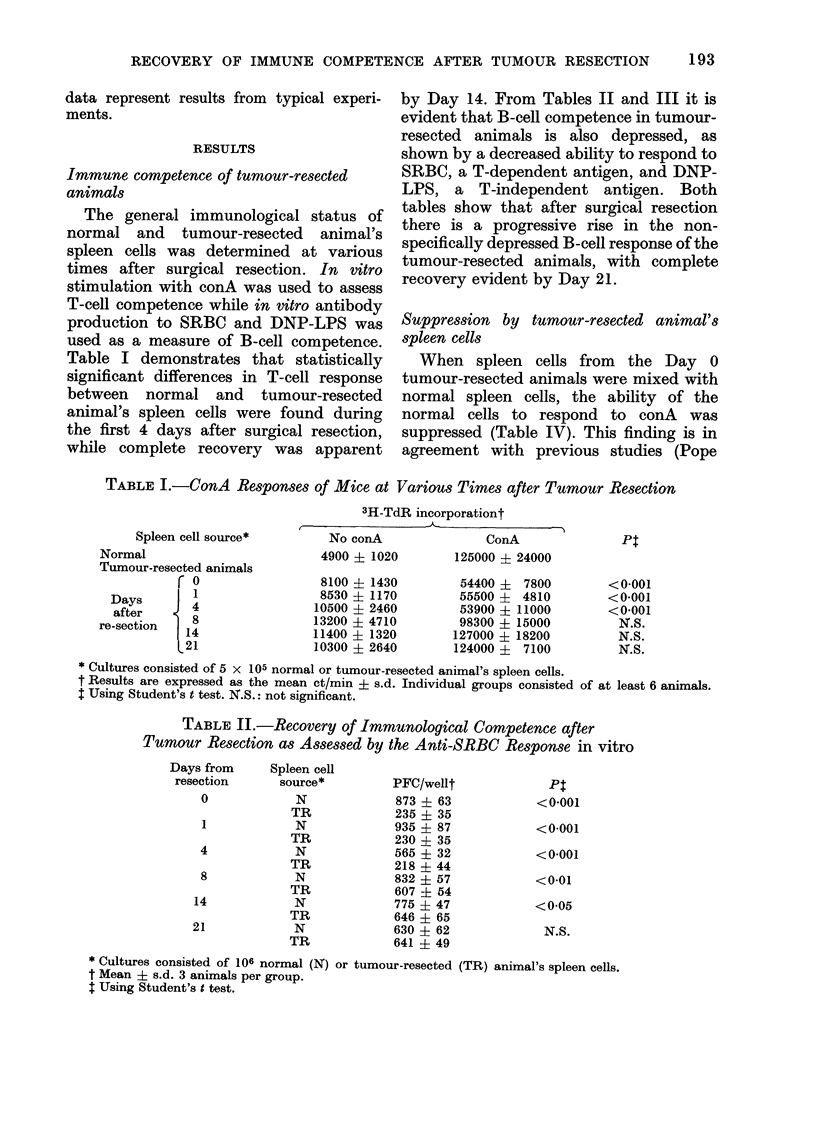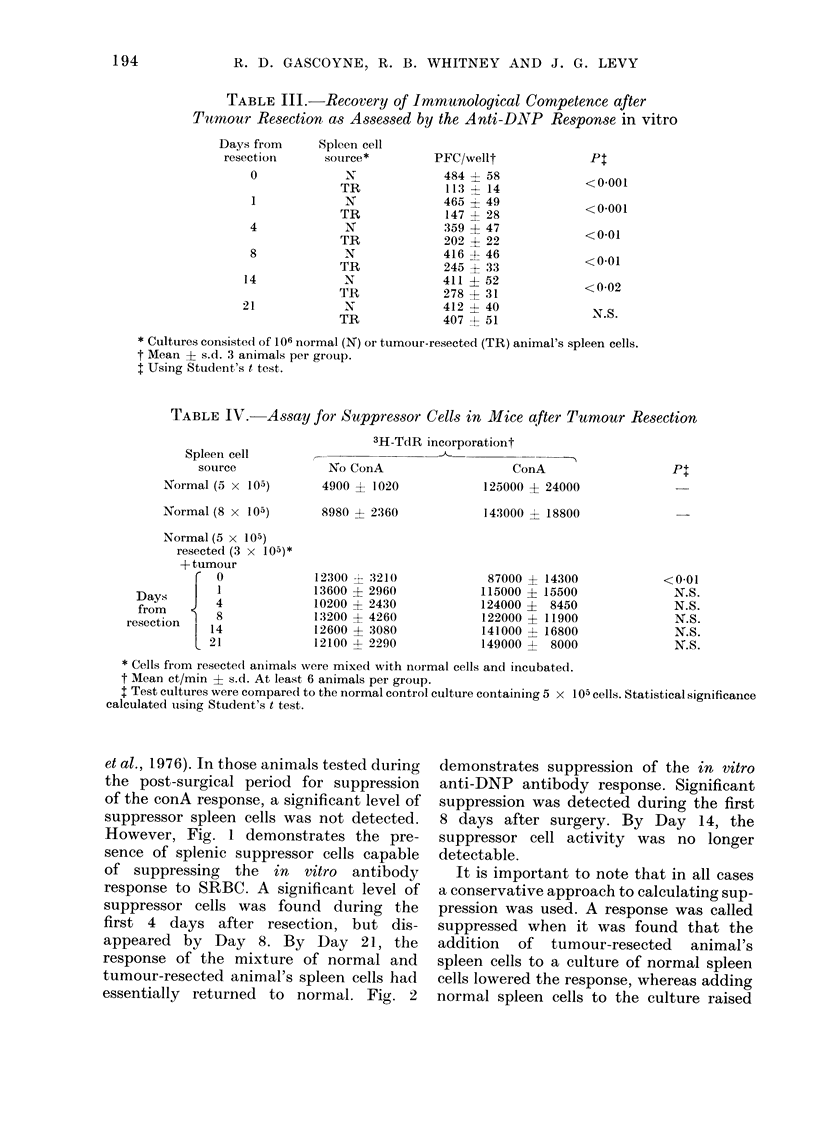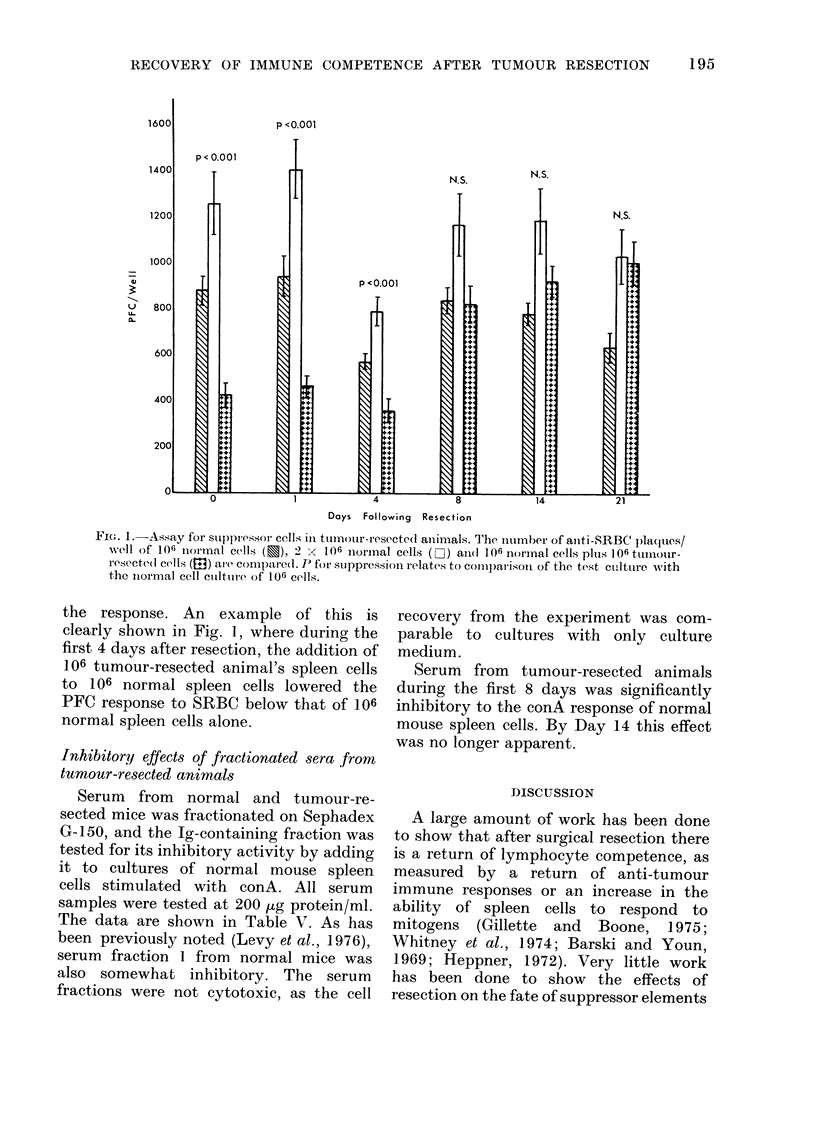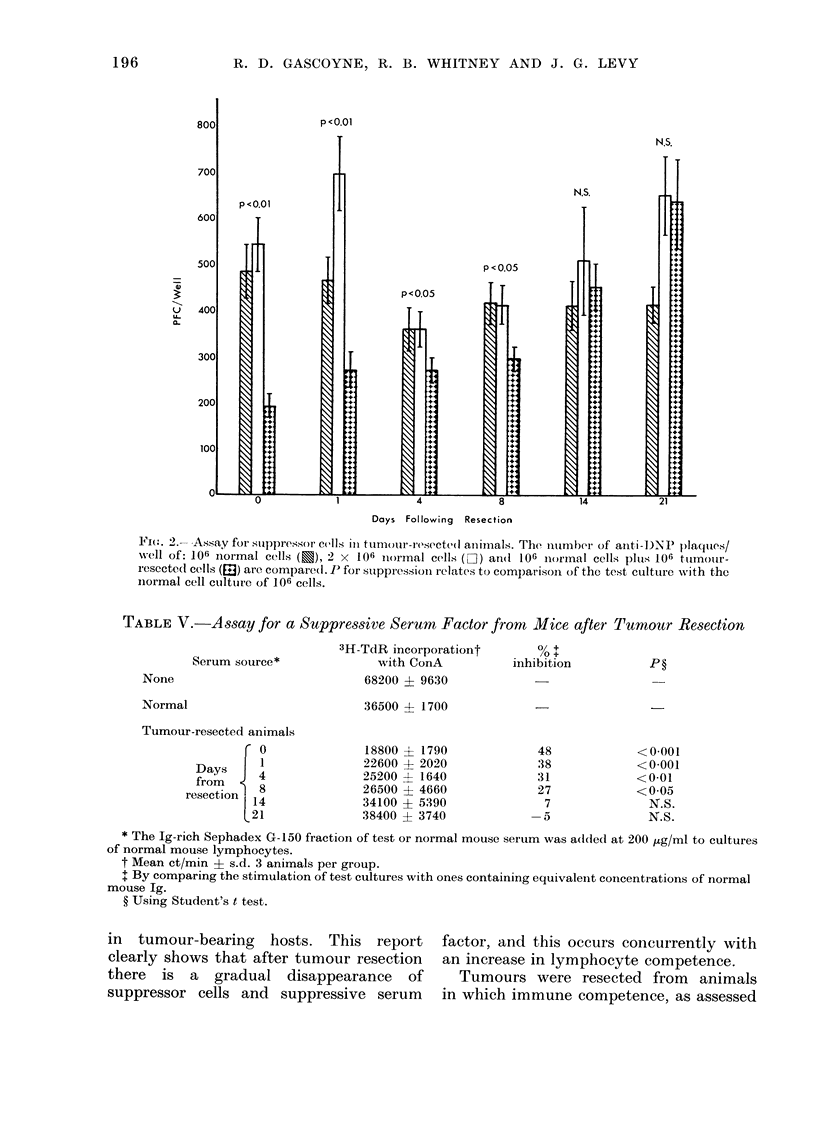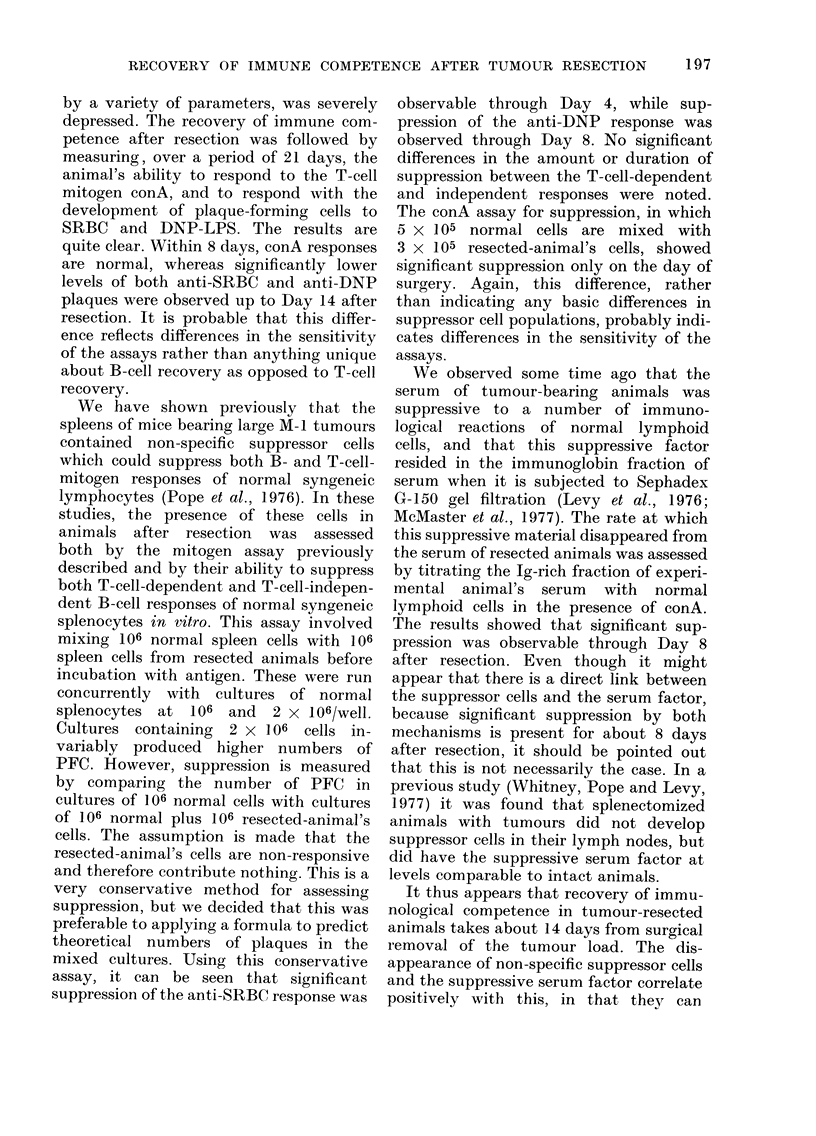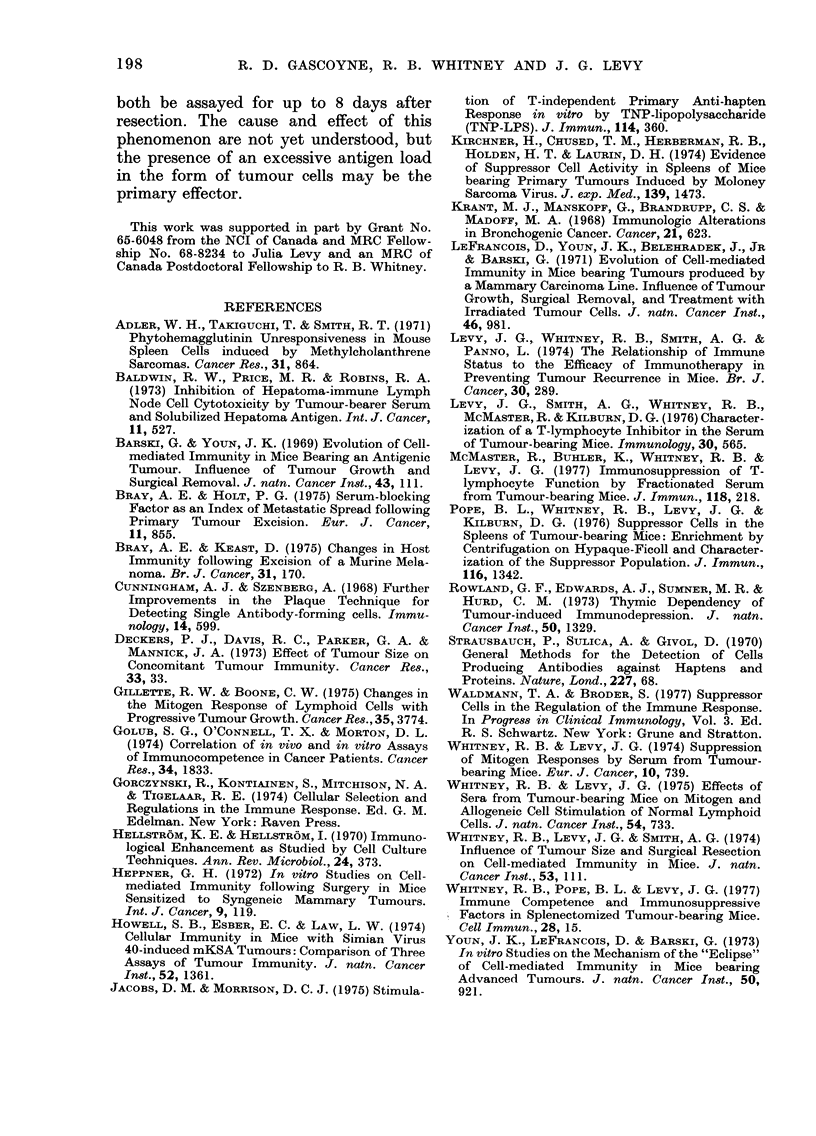# Recovery of immune competence after tumour resection in mice: correlation with loss of suppressor elements.

**DOI:** 10.1038/bjc.1978.27

**Published:** 1978-02

**Authors:** R. D. Gasocyne, R. B. Whitney, J. G. Levy

## Abstract

Changes in the immune competence and levels of suppressore elements were assessed by mitogen stimulation and in vitro antibody production, after resection of a transplantable sarcoma. Spleen cells from tumour-resected animals were found to have depressed responses to conA as well as to the antigens SRBC and DNP-LPS. This inability to respond was gradually overcome and, by Day 21 after resection, spleen cell competence had returned to normal levels. Suppressor cells isolated from the spleens of tumour-resected animals were capable of suppressing the conA response and PFC response of normal syngeneic spleen cells in vitro. The ability to suppress the conA response of normal cells disappeared by Day 1 after resection, while the ability to suppress the anti-SRBC and anti-DNP PFC response of normal cells disappeared by Day 8 and Day 14 respectively. Serum from tumour-resected mice was also found to be suppressive to the conA response of normal spleen cells. The inhibitory material responsible for suppression eluted with the Ig-containing fraction on Sephadex G-150. This inhibitory material gradually disappeared from the serum of tumour-resected mice and was no longer apparent by Day 14. Therefore, it appeared that the return of normal lymphocyte function after tumour-resection was concomitant with the disappearance of splenic suppressor cells and suppressive serum factor.


*Br. J. Cancer* (1978) **37**, 190

### RECOVERY OF IMMUNE COMPETENCE AFTER TUMOUR RESECTION IN MICE: CORRELATION WITH LOSS OF SUPPRESSOR ELEMENTS

R. D. GASCOYNE, R. B. WHITNEY AND J. G. LEVY

*From the Department of Microbiology, University of British Columbia, Vancouver, British Columbia, Canada V6T 1W5*

Received 1 September 1976 Accepted 24 October 1977

**Summary.—Changes in the immune competence and levels of suppressor elements were assessed by mitogen stimulation and *in vitro* antibody production, after resection of a transplantable sarcoma. Spleen cells from tumour-resected animals were found to have depressed responses to conA as well as to the antigens SRBC and DNP-LPS. This inability to respond was gradually overcome and, by Day 21 after resection, spleen cell competence had returned to normal levels. Suppressor cells isolated from the spleens of tumour-resected animals were capable of suppressing the conA response and PFC response of normal syngeneic spleen cells *in vitro*. The ability to suppress the conA response of normal cells disappeared by Day 1 after resection, while the ability to suppress the anti-SRBC and anti-DNP PFC response of normal cells disappeared by Day 8 and Day 14 respectively. Serum from tumour-resected mice was also found to be suppressive to the conA response of normal spleen cells. The inhibitory material responsible for suppression eluted with the Ig-containing fraction on Sephadex G-150. This inhibitory material gradually disappeared from the serum of tumour-resected mice and was no longer apparent by Day 14. Therefore, it appeared that the return of normal lymphocyte function after tumour-resection was concomitant with the disappearance of splenic suppressor cells and suppressive serum factor.**

PROGRESSIVE tumour growth in humans and laboratory animals is frequently coincident with progressive immunosuppression. Tumour-specific immune responses which develop during early tumour growth decline and often disappear entirely (Deckers *et al.*, 1973; Howell, Esber and Law, 1974; Youn, LeFrancois and Barski, 1973; Whitney, Levy and Smith, 1974). General immune responses, such as delayed hypersensitivity reactions to recall antigens and *in vitro* lymphocyte stimulation by mitogens and antigens, are also depressed in many cancer patients (Krant *et al.*, 1968; Golub, O'Connell and Morton, 1974). Similarly, the response of mouse spleen cells to both mitogens and specific antigens declines with increasing tumour size (Adler, Takiguchi and Smith, 1971; Rowland *et al.*, 1973). At present many immunological functions have been suggested to be involved in tumour growth. Recent theories have suggested that tumours grow progressively because of the presence of blocking factors, particularly blocking antibodies or antigen–antibody complexes (Baldwin, Price and Robins, 1973; Hellström and Hellström, 1970; Gorczynski *et al.*, 1974); because of a defect in the host's immune response, both cellular and humoral; or because of suppressor elements that develop concurrently with tumour growth and actively antagonize the host's response to tumours and other antigens (Kirchner *et al.*, 1974; Whitney and Levy, 1974; Waldmann and Broder, 1977).

Numerous time-course experiments describing the development of cell-mediated immunity in tumour-bearing hosts have shown that an "eclipse" of tumour-specific immunity occurs after the tumour reaches a certain critical size. In all cases, restoration of an immunologically responsive state followed surgical removal of the tumour (LeFrancois *et al.*, 1971; Heppner, 1972). Other work has shown that non-specifically depressed cell-mediated immunity in tumour-bearing hosts could be restored to normal levels after surgical resection (Gillette and Boone, 1975; Adler *et al.*, 1971). Loss of serum-blocking activity in tumour-bearing hosts following surgical removal of the tumour has also been well documented (Bray and Keast, 1975; Bray and Holt, 1975).

Previous studies in our laboratory have shown that the decrease in lymphocyte competence that occurs with progressive tumour growth was concomitant with the appearance of an immuno-suppressive serum factor which inhibited lymphocyte proliferation. This suppressive factor separated with the 7S immunoglobulins on Sephadex G150 and G-200, and could be removed by absorption on anti-mouse-Ig columns (Whitney and Levy, 1975; Levy *et al.*, 1976). It was also shown that mice with tumours developed suppressor cells in their spleens which inhibited mitogen responses of normal lymphocytes just as the serum factor did. Treatment of the suppressor spleen population with anti-$\theta$ or anti-Ig and complement did not remove the suppressor cells. In contrast, treatments which remove adherent cells, including passage through nylon wool columns and treatment with carbonyl iron, effectively removed the suppressor activity. It was concluded that the suppressor cell was of the macrophage/monocyte series (Pope *et al.*, 1976).

The present study was undertaken to follow the restoration of normal lymphocyte function after resection of solid tumours from DBA/2J mice. Recovery of normal function was correlated with the disappearance of the suppressive serum factor and splenic suppressor cells.

### MATERIALS AND METHODS

### *Experimental animals*

DBA/2J female mice (Jackson Laboratory, Bar Harbor, Maine) aged 8–10 weeks were used in all experiments.

### *Tumour*

The tumour used was a methycholanthrene-induced rhabdomyosarcoma (M-1) obtained originally from the Jackson Laboratory, and maintained both *in vivo* and *in vitro* in this laboratory for the past 4 years. Methods for the maintenance and culture of the tumour have been described elsewhere (Whitney *et al.*, 1974). Animals were injected with $2 \times 10^4$ viable M-1 cells and palpable tumours were observed 7–10 days later. Once palpable, the tumours grew uniformly. The tumour was grown s.c. on the animal's right abdominal side, thus allowing easy surgical resection.

### *Surgical resection*

Resections were carried out 32 days after tumour implantation. At this time, the tumour mass was 3·5–4·5 g. The animals were partially anaesthetized by i.p. injection of sodium pentobarbital (Nembutal—Abbott Laboratories, Montreal, Canada; 0·75 mg per animal) and were maintained under ether anaesthesia during surgical removal of the tumours. Previous studies in this laboratory had shown that surgical resection of small tumours, from mice in which immune competence had not yet become impaired, did not cause any change of immune status in resected animals in comparison to untreated controls (Levy *et al.*, 1974). Other studies had shown that sham-operated animals did not have suppressive factors in their serum (unpublished data). For these reasons, untreated normal animals were used in this study rather than sham-operated controls.

### *Preparation of lymphoid cell suspensions*

Normal and tumour-resected animal's spleens were removed aseptically, and cell suspensions were prepared by pressing them through stainless steel screens into phosphate-buffered saline (PBS), containing 5% foetal calf serum (FCS). The cells were centrifuged and resuspended in 10 ml of 0·83% $NH_4Cl$ for 3–4 min to remove erythrocytes, followed by centrifugation and one further wash with PBS. The cells were then counted by the trypan blue exclusion method.

*Assays for immune competence*

*Mitogen stimulation.*—Spleen cells were cultured in microtitre plates as has been previously described, with gentamycin (50 $\mu$g/ml) being the only antibiotic (Whitney *et al.*, 1974). The mitogen concanavalin A (conA) was used at a final concentration of 4 $\mu$g/ml. When testing for competence, the various cell populations were tested separately at $5 \times 10^5$ cells/well. When testing for suppressor cell activity $5 \times 10^5$ normal spleen cells were cultured with $3 \times 10^5$ tumour-resected animal's spleen cells. After 2 days in culture, $^3$H-thymidine was added (1 $\mu$Ci/well, sp. act. 2 Ci/mmol, Amersham/Searle, Don Mills, Ontario) and the cultures were harvested 18 h later, as previously described (Whitney and Levy, 1974).

In vitro *antibody production*

Spleen cells from normal and tumour-resected mice were cultured at $10^6$ and $2 \times 10^6$ per microtitre plate containing 0·25 ml RPMI-1640 medium supplemented with 10% FCS (Flow Laboratories, Lot No. 40551044) and $5 \times 10^{-5}$ M 2-mercaptoethanol. Antigens used were either $2{\cdot}5 \times 10^6$ SRBC/ml or 0·1 $\mu$g/ml DNP-LPS, the latter of which behaves *in vitro* as a T-cell-independent antigen. The DNP-LPS was prepared according to Jacobs and Morrison (1975). Cultures were incubated at 37°C on a rocking platform. The DNP-LPS cultures were incubated for 3 days and the SRBC cultures for 4 days, as preliminary assays had shown these to be the optimal times for detection of plaque-forming cells (PFC). The number of direct PFC was determined with a microscope-slide assay described previously (Cunningham and Szenberg, 1968). DNP plaques were determined using SRBC coated with dinitrophenylated rabbit anti-SRBC-Fab′ (Strausbauch, Sulica and Givol, 1970). Specific anti-DNP plaques were enumerated by substracting background SRBC plaques from the total number obtained. When testing for competence, normal and tumour-resected animal's spleen cells were cultured separately at $10^6$ cells/well. When testing for suppressor activity, $10^6$ normal spleen cells were cultured with $10^6$ tumour-resected animal's spleen cells. The viability of all cultures are about the same (36–48%). All results are expressed as PFC/well.

*Collection and preparation of serum*

Blood was taken from both normal and tumour-resected animals by exsanguination from the heart. It was allowed to clot overnight at 4°C, after which the serum was removed, centrifuged to remove residual erythrocytes, inactivated at 56°C for 30 min and stored at −20°C.

All work reported herein was carried out on the immunoglobulin-rich fraction of sera from a Sephadex G-150 column. Detailed procedures for this fractionation have been published previously (Levy *et al.*, 1976).

*Assay for inhibitory serum factor*

*Mitogen stimulation.*—Sera were assayed as previously described (Whitney *et al.*, 1974; Whitney and Levy, 1974, 1975). In brief, $5 \times 10^5$ normal spleen cells were cultured in 0·20 ml of RPMI-1640 medium supplemented with 2·5% FCS in microtitre plates with 200 $\mu$g/ml of test material from either normal or tumour-resected animal's serum. ConA was added in a volume of 0·05 ml at a concentration of 1 $\mu$g/ml. The cultures were incubated for 48 h, after which 1 $\mu$Ci of $^3$H-TdR was added. Cultures were harvested and $^3$H-TdR incorporation was measured 18 h later. Values achieved by cultures at equivalent quantities of normal mouse serum were taken as the 100% level, and percentage inhibition was calculated by comparing the uptake values of samples containing equivalent quantities of tumour-resected animal's serum to the 100% value, according to the formula:

$$\% \text{ inhibition} = \frac{\text{ConA response in cultures with NF1} - \text{ConA response in cultures with TF1}}{\text{ConA response in cultures with NF1}} \times 100$$

where NF1 = fractionated serum from normal mice, and

TF1 = fractionated serum from tumour-resected mice.

*Data presentation*

Where appropriate, results are expressed as the mean value ± s.d. The statistical significance of differences in mean values was determined by Student's *t* test. Differences were considered significant if the probability that the observed difference occurring by chance alone was <5% (*i.e.* $P<0{\cdot}05$). All data represent results from typical experiments.

RESULTS

*Immune competence of tumour-resected animals*

The general immunological status of normal and tumour-resected animal's spleen cells was determined at various times after surgical resection. *In vitro* stimulation with conA was used to assess T-cell competence while *in vitro* antibody production to SRBC and DNP-LPS was used as a measure of B-cell competence. Table I demonstrates that statistically significant differences in T-cell response between normal and tumour-resected animal's spleen cells were found during the first 4 days after surgical resection, while complete recovery was apparent by Day 14. From Tables II and III it is evident that B-cell competence in tumour-resected animals is also depressed, as shown by a decreased ability to respond to SRBC, a T-dependent antigen, and DNP-LPS, a T-independent antigen. Both tables show that after surgical resection there is a progressive rise in the non-specifically depressed B-cell response of the tumour-resected animals, with complete recovery evident by Day 21.

*Suppression by tumour-resected animal's spleen cells*

When spleen cells from the Day 0 tumour-resected animals were mixed with normal spleen cells, the ability of the normal cells to respond to conA was suppressed (Table IV). This finding is in agreement with previous studies (Pope

TABLE I.—*ConA Responses of Mice at Various Times after Tumour Resection*

| Spleen cell source* | | $^3$H-TdR incorporation† | | $P$‡ |
|---|---|---|---|---|
| | | No conA | ConA | |
| Normal | | 4900 ± 1020 | 125000 ± 24000 | |
| Tumour-resected animals | | | | |
| Days after re-section | 0 | 8100 ± 1430 | 54400 ± 7800 | <0·001 |
| | 1 | 8530 ± 1170 | 55500 ± 4810 | <0·001 |
| | 4 | 10500 ± 2460 | 53900 ± 11000 | <0·001 |
| | 8 | 13200 ± 4710 | 98300 ± 15000 | N.S. |
| | 14 | 11400 ± 1320 | 127000 ± 18200 | N.S. |
| | 21 | 10300 ± 2640 | 124000 ± 7100 | N.S. |

* Cultures consisted of 5 × $10^5$ normal or tumour-resected animal's spleen cells.
† Results are expressed as the mean ct/min ± s.d. Individual groups consisted of at least 6 animals.
‡ Using Student's *t* test. N.S.: not significant.

TABLE II.—*Recovery of Immunological Competence after Tumour Resection as Assessed by the Anti-SRBC Response* in vitro

| Days from resection | Spleen cell source* | PFC/well† | $P$‡ |
|---|---|---|---|
| 0 | N | 873 ± 63 | <0·001 |
| | TR | 235 ± 35 | |
| 1 | N | 935 ± 87 | <0·001 |
| | TR | 230 ± 35 | |
| 4 | N | 565 ± 32 | <0·001 |
| | TR | 218 ± 44 | |
| 8 | N | 832 ± 57 | <0·01 |
| | TR | 607 ± 54 | |
| 14 | N | 775 ± 47 | <0·05 |
| | TR | 646 ± 65 | |
| 21 | N | 630 ± 62 | N.S. |
| | TR | 641 ± 49 | |

* Cultures consisted of $10^6$ normal (N) or tumour-resected (TR) animal's spleen cells.
† Mean ± s.d. 3 animals per group.
‡ Using Student's *t* test.

TABLE III.—*Recovery of Immunological Competence after Tumour Resection as Assessed by the Anti-DNP Response* in vitro

| Days from resection | Spleen cell source* | PFC/well† | $P$‡ |
|---|---|---|---|
| 0 | N | 484 ± 58 | <0·001 |
| | TR | 113 ± 14 | |
| 1 | N | 465 ± 49 | <0·001 |
| | TR | 147 ± 28 | |
| 4 | N | 359 ± 47 | <0·01 |
| | TR | 202 ± 22 | |
| 8 | N | 416 ± 46 | <0·01 |
| | TR | 245 ± 33 | |
| 14 | N | 411 ± 52 | <0·02 |
| | TR | 278 ± 31 | |
| 21 | N | 412 ± 40 | N.S. |
| | TR | 407 ± 51 | |

* Cultures consisted of $10^6$ normal (N) or tumour-resected (TR) animal's spleen cells.
† Mean ± s.d. 3 animals per group.
‡ Using Student's $t$ test.

TABLE IV.—*Assay for Suppressor Cells in Mice after Tumour Resection*

| Spleen cell source | $^3$H-TdR incorporation† No ConA | $^3$H-TdR incorporation† ConA | $P$‡ |
|---|---|---|---|
| Normal ($5 \times 10^5$) | 4900 ± 1020 | 125000 ± 24000 | — |
| Normal ($8 \times 10^5$) | 8980 ± 2360 | 143000 ± 18800 | — |
| Normal ($5 \times 10^5$) + tumour resected ($3 \times 10^5$)* | | | |
| Days from resection 0 | 12300 ± 3210 | 87000 ± 14300 | <0·01 |
| 1 | 13600 ± 2960 | 115000 ± 15500 | N.S. |
| 4 | 10200 ± 2430 | 124000 ± 8450 | N.S. |
| 8 | 13200 ± 4260 | 122000 ± 11900 | N.S. |
| 14 | 12600 ± 3080 | 141000 ± 16800 | N.S. |
| 21 | 12100 ± 2290 | 149000 ± 8000 | N.S. |

* Cells from resected animals were mixed with normal cells and incubated.
† Mean ct/min ± s.d. At least 6 animals per group.
‡ Test cultures were compared to the normal control culture containing $5 \times 10^5$ cells. Statistical significance calculated using Student's $t$ test.

*et al.*, 1976). In those animals tested during the post-surgical period for suppression of the conA response, a significant level of suppressor spleen cells was not detected. However, Fig. 1 demonstrates the presence of splenic suppressor cells capable of suppressing the *in vitro* antibody response to SRBC. A significant level of suppressor cells was found during the first 4 days after resection, but disappeared by Day 8. By Day 21, the response of the mixture of normal and tumour-resected animal's spleen cells had essentially returned to normal. Fig. 2 demonstrates suppression of the *in vitro* anti-DNP antibody response. Significant suppression was detected during the first 8 days after surgery. By Day 14, the suppressor cell activity was no longer detectable.

It is important to note that in all cases a conservative approach to calculating suppression was used. A response was called suppressed when it was found that the addition of tumour-resected animal's spleen cells to a culture of normal spleen cells lowered the response, whereas adding normal spleen cells to the culture raised

FIG. 1.—Assay for suppressor cells in tumour-resected animals. The number of anti-SRBC plaques/well of $10^6$ normal cells (▧), $2 \times 10^6$ normal cells (□) and $10^6$ normal cells plus $10^6$ tumour-resected cells (⊞) are compared. *P* for suppression relates to comparison of the test culture with the normal cell culture of $10^6$ cells.

the response. An example of this is clearly shown in Fig. 1, where during the first 4 days after resection, the addition of $10^6$ tumour-resected animal's spleen cells to $10^6$ normal spleen cells lowered the PFC response to SRBC below that of $10^6$ normal spleen cells alone.

*Inhibitory effects of fractionated sera from tumour-resected animals*

Serum from normal and tumour-resected mice was fractionated on Sephadex G-150, and the Ig-containing fraction was tested for its inhibitory activity by adding it to cultures of normal mouse spleen cells stimulated with conA. All serum samples were tested at 200 $\mu$g protein/ml. The data are shown in Table V. As has been previously noted (Levy *et al.*, 1976), serum fraction 1 from normal mice was also somewhat inhibitory. The serum fractions were not cytotoxic, as the cell recovery from the experiment was comparable to cultures with only culture medium.

Serum from tumour-resected animals during the first 8 days was significantly inhibitory to the conA response of normal mouse spleen cells. By Day 14 this effect was no longer apparent.

### DISCUSSION

A large amount of work has been done to show that after surgical resection there is a return of lymphocyte competence, as measured by a return of anti-tumour immune responses or an increase in the ability of spleen cells to respond to mitogens (Gillette and Boone, 1975; Whitney *et al.*, 1974; Barski and Youn, 1969; Heppner, 1972). Very little work has been done to show the effects of resection on the fate of suppressor elements

FIG. 2.—Assay for suppressor cells in tumour-resected animals. The number of anti-DNP plaques/well of: $10^6$ normal cells (▧), $2 \times 10^6$ normal cells (□) and $10^6$ normal cells plus $10^6$ tumour-resected cells (⊞) are compared. $P$ for suppression relates to comparison of the test culture with the normal cell culture of $10^6$ cells.

TABLE V.—*Assay for a Suppressive Serum Factor from Mice after Tumour Resection*

| Serum source* | | $^3$H-TdR incorporation† with ConA | % ‡ inhibition | $P$§ |
|---|---|---|---|---|
| None | | 68200 ± 9630 | — | — |
| Normal | | 36500 ± 1700 | — | — |
| Tumour-resected animals | | | | |
| Days from resection | 0 | 18800 ± 1790 | 48 | <0·001 |
| | 1 | 22600 ± 2020 | 38 | <0·001 |
| | 4 | 25200 ± 1640 | 31 | <0·01 |
| | 8 | 26500 ± 4660 | 27 | <0·05 |
| | 14 | 34100 ± 5390 | 7 | N.S. |
| | 21 | 38400 ± 3740 | −5 | N.S. |

* The Ig-rich Sephadex G-150 fraction of test or normal mouse serum was added at 200 μg/ml to cultures of normal mouse lymphocytes.

† Mean ct/min ± s.d. 3 animals per group.

‡ By comparing the stimulation of test cultures with ones containing equivalent concentrations of normal mouse Ig.

§ Using Student's *t* test.

in tumour-bearing hosts. This report clearly shows that after tumour resection there is a gradual disappearance of suppressor cells and suppressive serum factor, and this occurs concurrently with an increase in lymphocyte competence.

Tumours were resected from animals in which immune competence, as assessed by a variety of parameters, was severely depressed. The recovery of immune competence after resection was followed by measuring, over a period of 21 days, the animal's ability to respond to the T-cell mitogen conA, and to respond with the development of plaque-forming cells to SRBC and DNP-LPS. The results are quite clear. Within 8 days, conA responses are normal, whereas significantly lower levels of both anti-SRBC and anti-DNP plaques were observed up to Day 14 after resection. It is probable that this difference reflects differences in the sensitivity of the assays rather than anything unique about B-cell recovery as opposed to T-cell recovery.

We have shown previously that the spleens of mice bearing large M-1 tumours contained non-specific suppressor cells which could suppress both B- and T-cell-mitogen responses of normal syngeneic lymphocytes (Pope *et al.*, 1976). In these studies, the presence of these cells in animals after resection was assessed both by the mitogen assay previously described and by their ability to suppress both T-cell-dependent and T-cell-independent B-cell responses of normal syngeneic splenocytes *in vitro*. This assay involved mixing $10^6$ normal spleen cells with $10^6$ spleen cells from resected animals before incubation with antigen. These were run concurrently with cultures of normal splenocytes at $10^6$ and $2 \times 10^6$/well. Cultures containing $2 \times 10^6$ cells invariably produced higher numbers of PFC. However, suppression is measured by comparing the number of PFC in cultures of $10^6$ normal cells with cultures of $10^6$ normal plus $10^6$ resected-animal's cells. The assumption is made that the resected-animal's cells are non-responsive and therefore contribute nothing. This is a very conservative method for assessing suppression, but we decided that this was preferable to applying a formula to predict theoretical numbers of plaques in the mixed cultures. Using this conservative assay, it can be seen that significant suppression of the anti-SRBC response was observable through Day 4, while suppression of the anti-DNP response was observed through Day 8. No significant differences in the amount or duration of suppression between the T-cell-dependent and independent responses were noted. The conA assay for suppression, in which $5 \times 10^5$ normal cells are mixed with $3 \times 10^5$ resected-animal's cells, showed significant suppression only on the day of surgery. Again, this difference, rather than indicating any basic differences in suppressor cell populations, probably indicates differences in the sensitivity of the assays.

We observed some time ago that the serum of tumour-bearing animals was suppressive to a number of immunological reactions of normal lymphoid cells, and that this suppressive factor resided in the immunoglobin fraction of serum when it is subjected to Sephadex G-150 gel filtration (Levy *et al.*, 1976; McMaster *et al.*, 1977). The rate at which this suppressive material disappeared from the serum of resected animals was assessed by titrating the Ig-rich fraction of experimental animal's serum with normal lymphoid cells in the presence of conA. The results showed that significant suppression was observable through Day 8 after resection. Even though it might appear that there is a direct link between the suppressor cells and the serum factor, because significant suppression by both mechanisms is present for about 8 days after resection, it should be pointed out that this is not necessarily the case. In a previous study (Whitney, Pope and Levy, 1977) it was found that splenectomized animals with tumours did not develop suppressor cells in their lymph nodes, but did have the suppressive serum factor at levels comparable to intact animals.

It thus appears that recovery of immunological competence in tumour-resected animals takes about 14 days from surgical removal of the tumour load. The disappearance of non-specific suppressor cells and the suppressive serum factor correlate positively with this, in that they can both be assayed for up to 8 days after resection. The cause and effect of this phenomenon are not yet understood, but the presence of an excessive antigen load in the form of tumour cells may be the primary effector.

This work was supported in part by Grant No. 65-6048 from the NCI of Canada and MRC Fellowship No. 68-8234 to Julia Levy and an MRC of Canada Postdoctoral Fellowship to R. B. Whitney.

### REFERENCES

ADLER, W. H., TAKIGUCHI, T. & SMITH, R. T. (1971) Phytohemagglutinin Unresponsiveness in Mouse Spleen Cells induced by Methylcholanthrene Sarcomas. *Cancer Res.*, **31**, 864.

BALDWIN, R. W., PRICE, M. R. & ROBINS, R. A. (1973) Inhibition of Hepatoma-immune Lymph Node Cell Cytotoxicity by Tumour-bearer Serum and Solubilized Hepatoma Antigen. *Int. J. Cancer*, **11**, 527.

BARSKI, G. & YOUN, J. K. (1969) Evolution of Cell-mediated Immunity in Mice Bearing an Antigenic Tumour. Influence of Tumour Growth and Surgical Removal. *J. natn. Cancer Inst.*, **43**, 111.

BRAY, A. E. & HOLT, P. G. (1975) Serum-blocking Factor as an Index of Metastatic Spread following Primary Tumour Excision. *Eur. J. Cancer*, **11**, 855.

BRAY, A. E. & KEAST, D. (1975) Changes in Host Immunity following Excision of a Murine Melanoma. *Br. J. Cancer*, **31**, 170.

CUNNINGHAM, A. J. & SZENBERG, A. (1968) Further Improvements in the Plaque Technique for Detecting Single Antibody-forming cells. *Immunology*, **14**, 599.

DECKERS, P. J., DAVIS, R. C., PARKER, G. A. & MANNICK, J. A. (1973) Effect of Tumour Size on Concomitant Tumour Immunity. *Cancer Res.*, **33**, 33.

GILLETTE, R. W. & BOONE, C. W. (1975) Changes in the Mitogen Response of Lymphoid Cells with Progressive Tumour Growth. *Cancer Res.*, **35**, 3774.

GOLUB, S. G., O'CONNELL, T. X. & MORTON, D. L. (1974) Correlation of *in vivo* and *in vitro* Assays of Immunocompetence in Cancer Patients. *Cancer Res.*, **34**, 1833.

GORCZYNSKI, R., KONTIAINEN, S., MITCHISON, N. A. & TIGELAAR, R. E. (1974) Cellular Selection and Regulations in the Immune Response. Ed. G. M. Edelman. New York: Raven Press.

HELLSTRÖM, K. E. & HELLSTRÖM, I. (1970) Immunological Enhancement as Studied by Cell Culture Techniques. *Ann. Rev. Microbiol.*, **24**, 373.

HEPPNER, G. H. (1972) *In vitro* Studies on Cell-mediated Immunity following Surgery in Mice Sensitized to Syngeneic Mammary Tumours. *Int. J. Cancer*, **9**, 119.

HOWELL, S. B., ESBER, E. C. & LAW, L. W. (1974) Cellular Immunity in Mice with Simian Virus 40-induced mKSA Tumours: Comparison of Three Assays of Tumour Immunity. *J. natn. Cancer Inst.*, **52**, 1361.

JACOBS, D. M. & MORRISON, D. C. J. (1975) Stimulation of T-independent Primary Anti-hapten Response *in vitro* by TNP-lipopolysaccharide (TNP-LPS). *J. Immun.*, **114**, 360.

KIRCHNER, H., CHUSED, T. M., HERBERMAN, R. B., HOLDEN, H. T. & LAURIN, D. H. (1974) Evidence of Suppressor Cell Activity in Spleens of Mice bearing Primary Tumours Induced by Moloney Sarcoma Virus. *J. exp. Med.*, **139**, 1473.

KRANT, M. J., MANSKOPF, G., BRANDRUPP, C. S. & MADOFF, M. A. (1968) Immunologic Alterations in Bronchogenic Cancer. *Cancer*, **21**, 623.

LEFRANCOIS, D., YOUN, J. K., BELEHRADEK, J., JR & BARSKI, G. (1971) Evolution of Cell-mediated Immunity in Mice bearing Tumours produced by a Mammary Carcinoma Line. Influence of Tumour Growth, Surgical Removal, and Treatment with Irradiated Tumour Cells. *J. natn. Cancer Inst.*, **46**, 981.

LEVY, J. G., WHITNEY, R. B., SMITH, A. G. & PANNO, L. (1974) The Relationship of Immune Status to the Efficacy of Immunotherapy in Preventing Tumour Recurrence in Mice. *Br. J. Cancer*, **30**, 289.

LEVY, J. G., SMITH, A. G., WHITNEY, R. B., MCMASTER, R. & KILBURN, D. G. (1976) Characterization of a T-lymphocyte Inhibitor in the Serum of Tumour-bearing Mice. *Immunology*, **30**, 565.

MCMASTER, R., BUHLER, K., WHITNEY, R. B. & LEVY, J. G. (1977) Immunosuppression of T-lymphocyte Function by Fractionated Serum from Tumour-bearing Mice. *J. Immun.*, **118**, 218.

POPE, B. L., WHITNEY, R. B., LEVY, J. G. & KILBURN, D. G. (1976) Suppressor Cells in the Spleens of Tumour-bearing Mice: Enrichment by Centrifugation on Hypaque-Ficoll and Characterization of the Suppressor Population. *J. Immun.*, **116**, 1342.

ROWLAND, G. F., EDWARDS, A. J., SUMNER, M. R. & HURD, C. M. (1973) Thymic Dependency of Tumour-induced Immunodepression. *J. natn. Cancer Inst.*, **50**, 1329.

STRAUSBAUCH, P., SULICA, A. & GIVOL, D. (1970) General Methods for the Detection of Cells Producing Antibodies against Haptens and Proteins. *Nature, Lond.*, **227**, 68.

WALDMANN, T. A. & BRODER, S. (1977) Suppressor Cells in the Regulation of the Immune Response. In *Progress in Clinical Immunology*, Vol. 3. Ed. R. S. Schwartz. New York: Grune and Stratton.

WHITNEY, R. B. & LEVY, J. G. (1974) Suppression of Mitogen Responses by Serum from Tumour-bearing Mice. *Eur. J. Cancer*, **10**, 739.

WHITNEY, R. B. & LEVY, J. G. (1975) Effects of Sera from Tumour-bearing Mice on Mitogen and Allogeneic Cell Stimulation of Normal Lymphoid Cells. *J. natn. Cancer Inst.*, **54**, 733.

WHITNEY, R. B., LEVY, J. G. & SMITH, A. G. (1974) Influence of Tumour Size and Surgical Resection on Cell-mediated Immunity in Mice. *J. natn. Cancer Inst.*, **53**, 111.

WHITNEY, R. B., POPE, B. L. & LEVY, J. G. (1977) Immune Competence and Immunosuppressive Factors in Splenectomized Tumour-bearing Mice. *Cell Immun.*, **28**, 15.

YOUN, J. K., LEFRANCOIS, D. & BARSKI, G. (1973) *In vitro* Studies on the Mechanism of the "Eclipse" of Cell-mediated Immunity in Mice bearing Advanced Tumours. *J. natn. Cancer Inst.*, **50**, 921.